# Metropolitan lizards? Urbanization gradient and the density of lagartixas (*Tropidurus hispidus*) in a tropical city

**DOI:** 10.1002/ece3.5518

**Published:** 2019-09-30

**Authors:** Antonio C. de Andrade

**Affiliations:** ^1^ Departamento de Engenharia e Meio Ambiente Universidade Federal da Paraiba Rio Tinto Brazil; ^2^ Centre of Urban Enviroments University of Toronto Mississauga Mississauga ON Canada

**Keywords:** Atlantic forest, land cover, reptiles, synurbic species, urban ecology

## Abstract

Urbanization, with its cohort of environmental stressors, has a dramatic effect on wildlife, causing loss of biodiversity and decline in population abundance customarily associated with increasing levels of impervious surface and fragmentation of native habitats. Some studies suggest that faunal species from open habitats, and with higher abundance in natural environments, seem more likely to tolerate and live in urban environments. Here I evaluate how the level of urbanization affects lagartixas (*Tropidurus hispidus*) one of the most common lizards found in open vegetation ecosystems in NE Brazil. I surveyed a total of 47 transects across sites that differed in proportion of impervious surface (high, mild, peri‐urban, and rural). I also collected specific biotic (herbaceous cover, tree, and arthropod abundance) and abiotic (amount of shelters and impervious surfaces) factors that could affect lagartixas abundance. Ants were the most common arthropod both in the rural and urban environment. Lagartixas thrive in urban environments, and trees and shelter were key predictors of their abundance. Lagartixas show a clear association with use of artificial structures. The low densities of lagartixas in rural areas and higher density in urbanized areas suggest that they colonized urban areas due to the hard surfaces and they probably are not exploiting a novel habitat, but somewhat responding to conditions resembling those in which they evolved. Finally, lagartixas are extremely common in tropical cities, they have a suite of features that are associated with selective pressures in cities and they might play a key functional role in urban ecosystems making this lizard an excellent system for the study of ecology and adaptation to the urban environments.

## Graphical abstract

Some hypothesis posits that faunal species from open habitats, and with higher abundance in nonurban environments, are more likely to tolerate the urban environments. The findings suggest that a broader environmental tolerance and ability to use man‐made structures are keys to survive in urban environment. Furthermore, *Tropidurus hispidus* is responding to conditions resembling those in which they evolved and this could explain their high density in tropical cities.

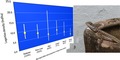

## 1 INTRODUCTION

The conversion of natural landscapes into urban areas replaces natural habitats with impervious surfaces, such as buildings and roads, causing them to become increasingly fragmented and isolated, resulting in loss of biodiversity and drastic changes in species' community composition (e.g., Aronson et al., 2014; Grimm et al., 2008; McKinney, 2002). Moreover, the structure of remnant vegetation is considerably reduced, particularly the shrub layer, and native species are replaced by exotic ones. Altogether these changes lead to a decrease in biodiversity (McKinney, 2006, 2008) that could result in reduced natural food resources for some species. For example, bird communities often shift to more granivorous species at the expense of insectivorous species in urban environments (Grimm et al., 2008). Thus, cities could have a negative impact and be a hostile habitat for many species.

Urbanization presents wildlife with a series of challenges, such as environmental stresses, competition with domestic/invasive species, noise, air, and light pollution (Birnie‐Gauvin, Peiman, Gallagher, Bruijn, & Cooke, 2016; Grimm et al., 2008). Nevertheless, it also grants wildlife with new ecological opportunities, shelter (e.g., holes and crevices in man‐made structures), lower predation rate, and access to abundant food, at least for some species, that can be a bonanza for life in the city (Francis & Chadwick, 2012; McKinney, 2002; Parris, 2016).

Many species might try to endure the dramatic changes in the environment brought about by urbanization; however, most will fail and only a few will thrive (McKinney, 2008; Shochat, Warren, Faeth, McIntyre, & Hope, 2006). Yet, those species that tolerate and exploit the unique conditions found in urban environments can become extremely abundant. These successful organisms (urban dwellers sensu FischerSchneider, Ahlers, & Miller, 2015) often attain higher densities in urban areas compared with rural environments (e.g., Francis & Chadwick, 2012; Møller et al., 2012; Shochat et al., 2006). However, the level of urbanization (e.g., amount of impervious surfaces) has an impact on the diversity and abundance of different organisms. For instance, the influence of biotic and abiotic factors changes according to the level of urbanization, causing fluctuations in species richness and abundance; in more urbanized areas, there is a reduction in diversity, while the total abundance of some species (usually non‐native species) increases (McKinney, 2006; Shochat et al., 2006).

The question of which factors influence species tolerance or aversion to urban life has long been contemplated. Over three decades ago, Diamond (1986) postulated that species capitalizing on city life were those able to use artificial structures, had a competitive advantage in relation to similar species, showed innovative behavior, and were dwellers of disturbed and open habitats. There are some indications that the attributes of successful city dwellers include high population densities in their ancestral rural habitats, superior competitive abilities over resources, a wide niche breadth, high fecundity, ability to withstand predators that are common in urban areas, the capability to use artificial substrates, and ability to exploit novel niches (Germaine & Wakeling, 2001; Møller et al., 2012; Rodda & Tyrrell, 2008; Shochat et al., 2010).

The combination of these traits, including behavioral flexibility and learning abilities, has been shown to be associated with urban tolerating species (Batabyal & Thaker, 2019; Callaghan et al., 2019; Littleford‐Colquhoun, Clemente, Whiting, Ortiz‐Barrientos, & Frere, 2017; Shochat et al., 2006; Sol, Gonzalez‐Lagos, Moreira, Maspons, & Lapiedra, 2014; Sol, Lapiedra, & Gonzalez‐Lagos, 2013; Winchell, Carlen, Puente‐Rolón, & Revell, 2018). This understanding, however, is limited as most studies have focused almost completely on birds and mammals (e.g., Chiari, Dinetti, Licciardello, Licitra, & Pautasso, 2010; Sol et al., 2014; Santini et al., 2019; but see Walsh, Goulet, Wong, & Chapple, 2018 and Winchell, Carlen, et al., 2018). Moreover, most studies are restricted to temperate regions (e.g., Beninde, Feldmeier, Veith, & Hochkirch, 2018; Møller et al., 2012), while the impact on most of the species of vertebrates that live in tropical cities is practically unknown; we have only a vague idea of how urbanization affects their ecology, morphology, and genetics.

Reptiles are a group that have been far less investigated in relation to specific urban features that might influence their tolerance or aversion of cities (review in French, Webb, Hudson, & Virgin, 2018). In recent years, however, a growing number of studies have explored how different aspects of urbanization affect lizard's biology (e.g., Kent et al., 2019) and one of the best studied groups is the New World *Anolis* lizards. For instance, urbanization causes extreme structural habitat changes that affect locomotor performance and drive shifts in limb morphology and body size in some *Anolis* species (Battles, Irschick, & Kolbe, 2019; Kolbe, Battles, & Avilés‐Rodríguez, 2016; Marnocha, Pollinger, & Smith, 2011; Winchell, Maayan, Fredette, & Revell, 2018; Winchell, Reynolds, Prado‐Irwin, Puente‐Rolón, & Revell, 2016), which might favor niche expansion (Battles, Moniz, & Kolbe, 2018). The urban environment also tends be hotter, with wider variation in temperature that could affect development, survival, and persistence of *Anolis* in cities (e.g., Battles & Kolbe, 2019; Hall & Warner, 2018; Tiatragul, Hall, Pavlik, & Warner, 2019). Nevertheless, we still have gaps in our knowledge. In South America, for instance, there is a dearth of studies evaluating how native lizard species respond to urbanization (e.g., de Andrade, Franzine, & Mesquita, 2019). Understanding how different species respond to urbanization is essential to expand our knowledge of key processes shaping urban wildlife communities.

Some authors suggest that higher density in nonurban areas could be one of the prerequisites to succeed in the urban environment (e.g., Chiari et al., 2010; Møller et al., 2012). Moreover, the urban habitats tend be more “open habitats” with trees and shrubs interspersed with open spaces and impervious surfaces, which could give an edge to native species that exploit similar nonurban environments (e.g., Diamond, 1986). In Brazil, one of the most common lizards in open vegetation ecosystems is *Tropidurus* spp. that can be found both in rock and open ground environments (Carvalho, 2013; Oliveira, Pereira‐Ribeiro, Winck, & Rocha, 2019) making it a good candidate to succeed in the urban environment.

In the area where I carried out this study, one of the most abundant native lizards in nonurban open vegetation areas is *Tropidurus hispidus* (Carvalho, 2013; Freire, 1996). Here I assess how degree of urbanization impacts *T. hispidus* (lagartixas). The more‐individuals hypothesis postulates that species with larger populations have decreasing chances of local extinction, facilitating their occurrence in urban environments (Chiari et al., 2010). Therefore, I predict lagartixas will be successful in the urban environment.

Artificial substrates and the smooth surfaces of buildings and walls are a hallmark of the urban environment and present a series of locomotor challenge for lizards that could drive adaptive morphological changes in urban populations (e.g., Kolbe, Battles, et al., 2016; Marnocha et al., 2011). Urban populations of *A. cristatellus*, in their native range, have longer limbs relative to body size in urban populations, allowing it to sprint faster and to be more stable on smooth, vertical substrates (Winchell, Maayan, et al., 2018; Winchell et al., 2016). This species also has a preference for using artificial substrates in urban environments, which could lead to increased exploitation of man‐made habitats and higher abundance in urban environments (Winchell, Carlen, et al., 2018). Some studies indicate that lizard species more abundant in urban areas tend to use artificial substrates more often (Germaine & Wakeling, 2001; Koenig, Shine, & Shea, 2001; Winchell, Carlen, et al., 2018); thus, I expect lagartixas to use artificial structures regularly. Since in the urban environment there is an ecological release, with lower predation pressure and higher availability of food (Shochat et al., 2010, 2006), I predict the density of lizards to be higher in urban environment in relation to nonurban areas.

## 2 METHODS

### 2.1 Study species

**Figure 1:**
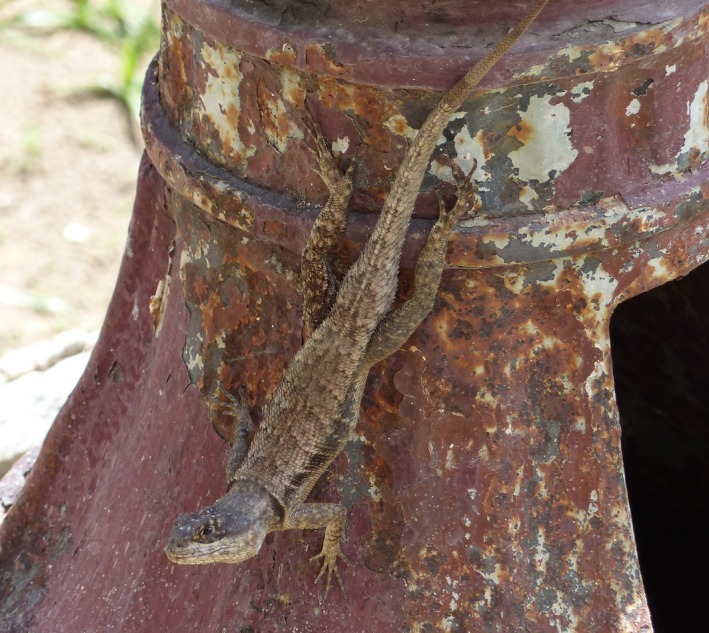
An adult male of lagartixas perching on an artificial substrate, also used as shelter

*Tropidurus hispidus* (Figure 1) is a terrestrial, medium‐sized lizard; the maximum snout–vent length (SVL) range for males is 109–143 and 89–91 mm for females (Albuquerque, Protazio, Cavalcanti, Lopez, & Mesquita, 2018). This species has a wide range distribution in open habitats of South America and shows a continuous and uniform distribution in the Caatinga dry forest and coastal areas of northeastern Brazil (Carvalho, 2013). *Tropidurus hispidus* lizards are a sit‐and‐wait forager that feeds on a diverse array of arthropod, mostly ants and other insects, but also eats leaves and fruits (Carvalho, 2013; Ribeiro & Freire, 2011).

### 2.2 Study area

**Figure 2:**
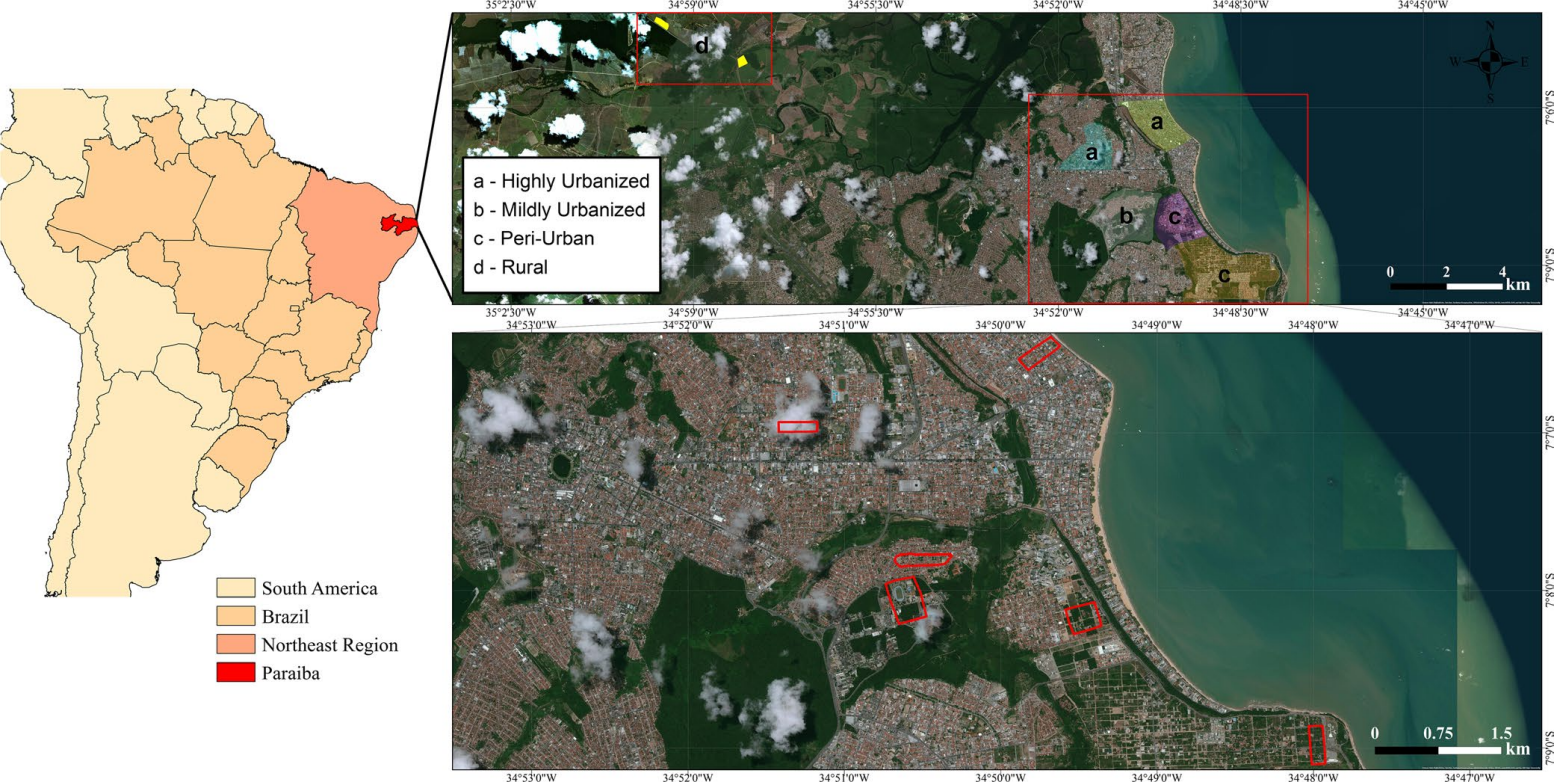
Maps showing the location of the districts selected, and urbanization categories, for the survey. The left panel highlights Paraiba state and NE Brazil, the top panel the selected districts, note on the upper left the rural sites. The lower panel shows the urban sites surveyed in Joao Pessoa city

Using available data on number of buildings, residences and population density (Sposati et al., 2009), and Google Earth to evaluate the level of urbanization (proportion of impervious surface; e.g., houses, buildings, roads, parking lots and other infrastructures), I selected five districts in the city of Joao Pessoa ( >800,000 people), capital of Paraiba, northeastern Brazil (Figure 2), with decreasing levels of urbanization: Manaira (approximately 256 ha; proportion of impervious surface >90%), Bairro dos Estados (area: 172 ha, impervious surface >80%), Castelo Branco (372 ha; impervious surface approximately 60%), Altiplano (229 ha and approximately 48.9% covered with impervious surface), and Portal do Sol (569 ha; 46.8% covered with impervious surface). Manaira and Bairro Estados are the districts with highest proportion of buildings, while Castelo Branco is a residential area and the other two districts are a mix of residential areas, small farms, and conservation areas (Sposati et al., 2009).

Based on those data, I selected a total of eight sites in the following gradients of urbanization (two sites per category): (a) highly urbanized, where the proportion of impervious surface (pavement, asphalt, buildings) is equal or higher than 70%; (b) mildly urbanized, areas with <70% but more than 50% impervious surface; (c) peri‐urban at the fringe of urban expansion, with <50% covered by impervious surface and ≥30% covered by native vegetation (Atlantic forest); and (d) rural sites outside the city, with <5% covered by impervious surface and ≥40% covered by crops (sugarcane) or other vegetation. The rural sites were located at about 8 and 12 Km N from Joao Pessoa (Figure 2). The proportion of canopy cover (native vegetation or orchard) and open vegetation in these sites range from about 18% (Rural 1) to 7% (Rural 2). Since the urban sites showed differences in the amount of vehicular traffic and green areas, I evaluated (using Google Earth) the local proportion of area that is impervious surface in each site where the surveys were carried out (Table 1 and Figure 2). The annual mean temperature in the area is 25°C and shows little variation during the year. The rainy season occurs from March to July, and annual rainfall ranges from 1,500 to 1,700 mm (Lima & Heckendorff, 1985).

**Table 1:** Details of urbanization in the surveyed sites

Sites	Area covered by “transects” (ha)	% Impervious surface	Type of use	Amount of car traffic	Descriptor
Estados	5.1	93.1	Buildings, residential, commerce	High	Highly urbanized
Manaira	7.6	90.5	Buildings, residential, commerce	High	Highly urbanized
Castelo	7.9	73.1	Residential	Low	Mildly urbanized
Ufpb	20	65.8	University	Medium	Mildly urbanized
Paco Leoes (Altiplano)	10.6	16.9	Residential, small farms	Low	Peri‐urban
Estacao (Portal do Sol)	7.3	13.9	Residential, small farms	Low/none	Peri‐urban
Rural 1	10	1.4	Small farms, sugarcane crops	Low	Rural
Rural 2	11.8	<1	Sugarcane crop	Low/none	Rural

### 2.3 Lizard survey

I conducted lizard surveys at a total of 36 randomly chosen urban “transects” (streets) of unequal length (range = 95–155 m), 12 for each urbanization category. In the rural areas, I surveyed a total of 11 unpaved roads/trails (length range = 90–150 m): 5 in one site and 6 in the other site. I carried out the survey between December 2018 and January 2019. I surveyed each transect four times, two times each between 8:00–11:00 hr and 14:00–16:00 hr, with at least 2‐day interval between surveys. Survey times were selected to span the maximum activity period of lizards. When temperatures are too high (midday), the lagartixas move to shaded substrate or become inactive in shelters. I did not conduct survey on rainy days. Each “transect” was walked at a speed of about 1 km/hr, and when a lizard was detected, I recorded the distance to observer (sighting distance), the angle in relation to the trail (used for the estimation of density), and height they were perched/located (to evaluate the use of vertical space). Distance was recorded with a Bushnell 4 × 21 mm Laser Rangefinder.

### 2.4 Data on microhabitat

After the surveys, I sampled the microhabitats along each “transect” (street/road), in terms of impervious cover, herbaceous vegetation cover, number of shelters (e.g., crevices in wall or pavement, holes, and logs), and number of arthropods. For this sampling, I used 2 × 2 m quadrats that were placed in the beginning, middle, and end of each transect, and I flipped a coin to choose on which side of the transect to place the quadrat. I visually estimated the proportion of impervious surface (pavement) and herbaceous vegetation and counted the number of shelters (only those with dimensions to accommodate an adult lizard) inside the quadrats. To evaluate abundance of arthropods, I delimitated a 0.5 × 0.5 m area in each quadrat, and for 30 s, I scanned and counted all insects and other arthropods. I also counted all trees (with Diameter at Breast Height ≥10 cm) in each “transect” (street or road). As the transects had differences in length, I corrected the number of trees by dividing it by the transect length and I used this rate (trees/m) for comparisons.

### 2.5 Data analyses

I used the software Distance 7.2 (Thomas et al., 2010) to calculate the lizard density. The distance sampling analyses fit a detection function to the observed distance distribution, and this fitted function is used to estimate the proportion of individuals in the area. Distance sampling has been shown to give accurate and unbiased estimates of population density (Buckland et al., 2001; Thomas et al., 2010). I assessed the following combinations of functions and adjustment terms as suggested by Thomas et al. (2010): uniform key with cosine adjustments; half‐normal key with cosine; half‐normal key with hermite polynomial adjustments; and harzard rate with simple polynomial adjustments. Following Thomas et al. (2010), I truncated the data in the furthest 6% of distances to delete outliers and improve model fit. Since one of the surveyed areas had a low number of sightings (<30), I also tested models in which the detection function was pooled across strata (estimated globally) or for each stratum separately. I selected the best detection function that fitted the data by comparing the detection function histograms and by checking the goodness‐of‐fit statistics. Both models (globally and by stratum) produced reasonable fits, and I selected the best model (i.e., detection function estimated globally) based on the lowest Akaike's information criterion (Buckland et al., 2001).

To compare density between areas with different levels of urbanization, I examined the degree of overlap between confidence interval (Cumming & Finch, 2005). A lack of overlap in the 95% CI is correspondent to a chance event with *p* = .01 (Cumming, 2009; MacGregor‐Fors & Payton, 2013).

To determine the influence of environmental variables (biotic and abiotic) on lagartixas abundance, I used multiple linear regression. Since high correlation between explanatory variables can lead to wrong conclusions, I determined the collinearity of predictors (impervious surface, herbaceous vegetation, shelters, number of arthropods, and trees/m) using Pearson's correlation test. When pairs of variables were highly correlated (*r* > .7), I removed one of the variables from the analysis. The variable mean herbaceous cover was excluded from the analyses because of a high negative level of correlation with impervious surface (*r* = −.85).

I tested the assumption of autocorrelation of residuals with the Durbin–Watson test and obtained a value of 1.98. The assumption of independence was met, since the Durbin–Watson statistic is close to 2. The values of this test statistic vary between 0 and 4, with a value close to 2 meaning that the residuals are uncorrelated (Field, 2009).

Although the use of stepwise multiple regression is a common practice in ecology, it has a series of shortcomings that could lead to erroneous conclusions (Whittingham, Stephens, Bradbury, & Freckleton, 2006). Thus, I forced all predictors into the model simultaneously. I followed the recommendations of Field (2009) for the regression analyses and used SPSS v 13 to carry out the analyses.

## 3 RESULTS

I recorded a total of 234 lagartixas sightings during the surveys performed in the urban and rural areas; this sample size allows accurate estimates of density (Buckland et al., 2001). The only other species of lizard I sighted during the surveys were the teiid lizards *Ameiva ameiva* and *Tupinambis merianae*, but just once in the peri‐urban sites, and whiptails (*Cnemidophorus ocellifer*) in the peri‐urban (*n* = 5) and rural sites (*n* = 18). The small sample size for teiids (*n* < 40) precludes the use of distance sampling to estimate their density. The model with a hazard rate key function and simple polynomial adjustment fitted the data best and resulted in densities ranging from 1.79 lizards/ ha in rural area to 8.72 lizards/ ha in peri‐urban areas (Table 1). The density of lagartixas was lower at rural sites than at peri‐urban and medium urbanized areas. The 95% confidence intervals for density do not overlap (Table 2), a difference significant at *p* ≤ .01.

**Table 2:** Densities of the lagartixa at different levels of urbanization

Level of urbanization	Density (95% CI)	%CV	*N*	Sampling effort (total km walked)
High	4.5 (2.5–8.2)	28.	43	5.9
Medium	6.89 (3.8–12.45)	27.6	69	6.2
Peri‐urban	8.7 (6.5–11.68)	13.87	93	6.6
Rural	1.79 (0.86–3.7)	34.1	15	5.2

Note

Density represents individuals/ha. *N* is the number of individuals after truncating the furthest distances (see Section 2.5).

Abbreviations: CI, confidence interval; CV, coefficient of variation.

**Figure 3:**
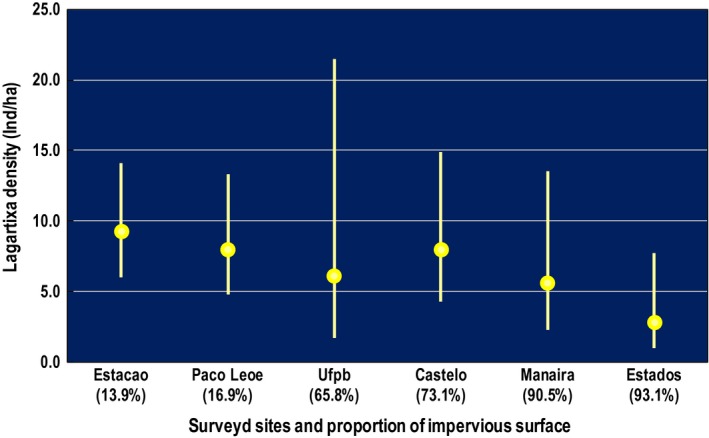
Density of lagartixas in relation to proportion of impervious surface (see Table 1 for further details) in the urban areas. The breakdown by sites reduces the number of transects to 6 per site, which is not the most adequate for estimation of density since a lower number of transects (<10) reduce the precision of estimates (Buckland et al., 2001). Rural sites were not included due to reduced number of sightings per site. Dots show mean and lines 95% CI

The urban sites were paired according to degree of urbanization, but they showed subtle differences in the amount of vehicular traffic, impervious surface, presence of potential predators (e.g., cats), and amount of vegetation. Thus, I also breakdown the density estimates by site. The differences between sites apparently had a more visible effect only in the mildly urbanized UFPB site and the highly urbanized Estados site (Figure 3) that had the highest variability in density (CV = 52.5% and 41.3%, respectively).

**Figure 4:**
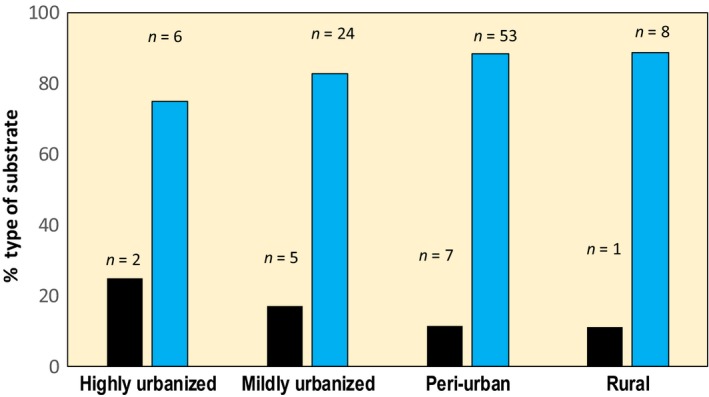
Percentage of types of substrate (black bar = natural, blue bar = artificial substrate) used by lagartixas when perched off the ground within each category of urbanization. The chi‐square goodness‐of‐fit tests revealed significant differences in the mildly urbanized (*χ* ^2^ = 12.45; *df* = 1; *p* < .001) and peri‐urban sites (*χ* ^2^ = 35.3; *df* = 1; *p* < .001). In the other sites, the *n* < 5 in one of the cells precluded the test

During the survey, I regularly sighted lagartixas on the ground (54.7%, *n* = 234). When not on the ground, the lagartixas were often sighted on artificial substrate such as walls and construction materials (Figure 4) and they used significantly more artificial substrate than natural (*χ* ^2^ = 54.5; *df* = 1; *p* < .001). Lagartixas perched off the ground at heights lower than 7 m (mean 1.9 m ± 1.4 *SD*; range: 0.2–6 m) and most commonly at height between 1 and 3 m (83.9%, *n* = 106).

**Figure 5:**
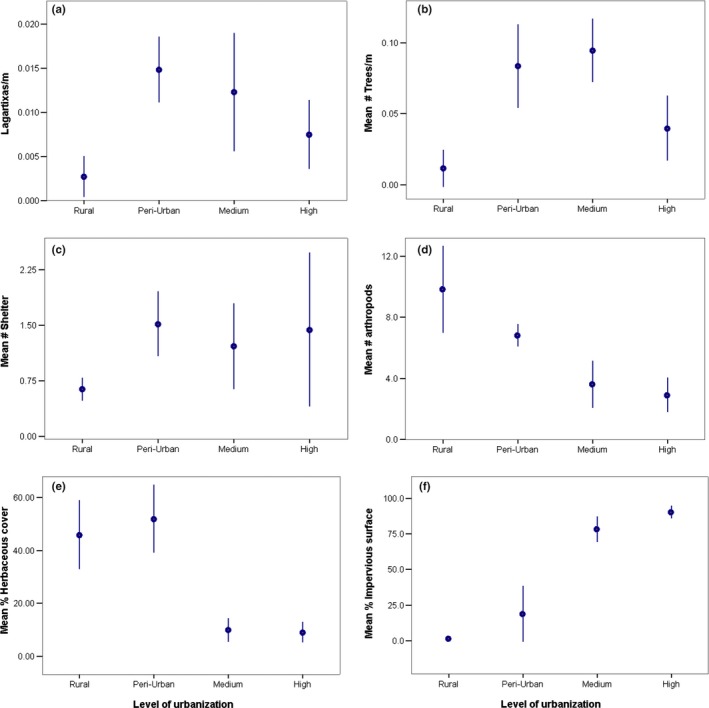
Difference in lagartixas abundance (a) and variability in biotic (b, d, e) and abiotic variables (c, f) according to the level of urbanization. Dots show mean, and lines represent 95% CI

There was variation in the different biotic and abiotic variables depending on the level of urbanization (Figure 5b–f). The proportion of herbaceous cover and the abundance of arthropods in the transects were higher in less urbanized areas (Figure 5d,e). Ants, the most important prey category for lagartixas, were the most common arthropod scanned both in the rural (95.2%, *n* = 334) and urban areas (95.5%, *n* = 488).

The regression model explained 37.5% (*R* ^2^) of the variability in lagartixa abundance and predicted lagartixas abundance significantly well (*F*_4, 42_ = 6.3, *p* < .0001). The variables shelter and trees made a significant contribution (*p* < .05) to predicting lagartixa abundance, whereas abundance of arthropods had no impact (Table 3).

**Table 3:** Effect of environmental variables on lagartixas abundance

	*B*	*SE*	*β*	*t*	*p*
Intercept	0.003	0.004		0.787	.44
Mean # Arthropods	−8.29E‐005	0.000	−0.037	−0.217	.83
Mean % impervious surface	−5.40E‐006	0.000	−0.027	−0.158	.87
Mean # Shelters	0.003	0.001	0.423	3.153	.003
Trees/m	0.052	0.023	0.302	2.22	.032

## 4 DISCUSSION

The results provide evidence that urbanization has a positive effect on the density of lagartixas. The peri‐urban and medium urbanized areas had the highest density of lagartixas, whereas the lowest densities were in the rural sites, despite a higher abundance of ants (see Figure 5d), an important food resource for lagartixas (Albuquerque et al., 2018; Ribeiro & Freire, 2011). The density of lagartixas in the highly urbanized site, even though higher, did not differ significantly from the rural areas. These results suggest quality of available habitat and specific aspects of urbanization, rather than degree of urbanization, may affect density.

It is possible that in the rural sites lagartixas were at disadvantage competing with other lizards such as whiptails; thus, the higher density in urban area could be the result of a relaxation in competition (Germaine & Wakeling, 2001; Shochat et al., 2010, 2006) benefiting lagartixas. The importance of interspecific competition regulating lizard abundance in urban areas is illustrated by the invasive *Anolis cristatellus* in Miami, where it attains significantly higher abundance at sites that lack species with a similar niche (Kolbe, VanMiddlesworth, et al., 2016). Nevertheless, the occurrence of whiptails and other teeid lizards in the peri‐urban sites, which had the highest densities of lagartixas, undermines this possibility for lagartixas.

The multiple linear regression model suggests that trees and shelter were key predictors of lagartixa abundance. The positive association of lagartixas with shelter and trees might be the result of individuals selecting microhabitats that provide critical refuge for avoiding predators, and these places could also guarantee optimum sites for laying eggs and for sheltering young individuals. Differences in egg and youngsters survival could contribute to variation in density across the urban gradient (Tiatragul et al., 2019). Another nonmutually exclusive possibility is that the presence of shelter and trees could provide shaded areas for thermoregulation.

Cities are warmer when compared with natural habitats (Ackley, Angilletta, DeNardo, Sullivan, & Wu, 2015; Grimm et al., 2008; Parris, 2016), and increased urban temperatures could have direct impact on lizards' fitness (Battles & Kolbe, 2019; Hall & Warner, 2018). Higher temperatures increase the body temperature of lizards, which could probably involve risk of overheating and increased thermoregulatory costs in the hotter urban environment (Battles & Kolbe, 2019; Kearney, Shine, & Porter, 2009). Moreover, extreme temperatures in urban environments increase egg mortality and might have negative impact on lizard development (Hall & Warner, 2018). Unfortunately, I did not measure local temperature and this abiotic variable might have had some influence on the results.

Lagartixas often used artificial substrates as perches, refuge, or sites for foraging. This could be an important aspect of their ability to thrive in urban areas. For example, in the peri‐urban areas, when perching off the ground, in over 61% of sightings they were usually in walls suggesting the presence of infrastructure and artificial surfaces providing refuges could be critical for their occurrence. Urban *Anolis* species show striking differences in use of artificial substrates; the most abundant species uses artificial substrates more often and seems much more tolerant to the stress associated with the artificial hard surfaces in cities (Winchell, Carlen, et al., 2018). Likewise, lizards from urban environments in temperate climates that are able to use artificial substrates also seem to do well in cities (e.g., Koenig et al., 2001; Littleford‐Colquhoun et al., 2017; Prosser, Hudson, & Thompson, 2006).

It is remarkable that in the rural sites, I consistently found lagartixas along artificial substrates; in some trails, they were absent and yet at about 100 m on the freeway, on a 1‐m‐height wall in the median strip (not surveyed), they were regularly seen for kilometers at a time. Even when in the middle of a sugarcane plantation, over 3 km far from the freeway, I could see lagartixas only in proximity to artificial structures (e.g., concrete wall of small dams). Carvalho et al. (2016) noted that the occurrence of a population of *T. hispidus*, in one southern area of its distribution, apparently was opportunistic and associated with man‐made structures. This raises the possibility that lagartixas might profit from the anthropogenic built landscape by experiencing not only substantial population growth, but also range expansion.

The tropical forest and pasture are efficient barriers for lagartixas, and paved highways or roads might be used as corridors for the colonization of adequate habitats (Vitt, Zani, & Caldwell, 1996). The use of suitable anthropogenic structures for dispersal, and gene flow, has been shown in urban lizards (Beninde et al., 2018, 2016). These artificial corridors used by lagartixas could be seen as kind of freeway for gene flow among populations and could be homogenizing allele frequencies and eroding differentiation among populations. This possibility is speculative, but deserves further studies.

## 5 A CITY SLICKER LIZARD

Despite the asphalt and concrete surfaces, the traffic pollution and an abundance of potential predators (e.g., cats) lagartixas are more common in urban environments than in more natural settings. Artificial substrates, or hardscapes, are ubiquitous in cities and could be seen as equivalent to natural rock outcrops and cliffs (Richardson, Lundholm, & Larson, 2010). Thus, the urban environment resembles the open habitats with rocks outcrops, where these lagartixas probably evolved (Carvalho, 2013), hence their high abundance in urban environments. This possibility has been proposed for other urban species such as birds that use the hardscape as surrogate for their native habitats (e.g., Erz, 1966). The high urban densities of lagartixas probably are due to the abundance of hardscape habitats in the city and their relative paucity in rural areas. The low densities in rural areas, and association with man‐made structures, and higher density in urbanized areas are suggestive that lagartixas colonized urban areas due to the hard surfaces. Thus, they probably are not exploiting a novel habitat (e.g., Ducatez, Sayol, Sol, & Lefebvre, 2018), but somewhat responding to conditions resembling those in which they evolved (Francis & Chadwick, 2012).

The results indicate that lagartixas are urban dwellers (sensu Fischer et al., 2015). Generalist species, with broad environmental tolerance, apparently are able to exploit novel habitats and succeed under the multiple environmental and ecological scenarios that are common in urban ecosystems (Bonier, Martin, & Wingfield, 2007; Callaghan et al., 2019; Ducatez et al., 2018; Winchell, Carlen, et al., 2018). This raises the possibility of a rapid adaptive evolution resulting in increased fitness and facilitating urban life (Johnson & Munshi‐South, 2017; Littleford‐Colquhoun et al., 2017; Winchell, Maayan, et al., 2018; Winchell et al., 2016). Whether lagartixas are adapting to the extreme selection in cities remains unknown, however.

## 6 LAGARTIXA AS MODEL SYSTEM TO STUDY ECOLOGY AND SELECTIVE PRESSURES IN CITIES

Lagartixas likely play an important role in trophic interactions and terrestrial ecosystem function in urban environments because of their high abundances (El‐Sabaawi, 2018; Winfree, Fox, Williams, Reilly, & Cariveau, 2015). For instance, lizards are an important food resource for many tropical bird species (Poulin et al., 2001) and lagartixas might be a key resource for birds in urban environments.

Basic ecological and evolutionary mechanisms in urban ecosystems remain poorly understood, and most studies are limited to temperate regions (Alberti et al., 2016; El‐Sabaawi, 2018; Faeth, Bang, & Saari, 2011; Johnson & Munshi‐South, 2017; McKinney, 2008; Santangelo, Rivkin, & Johnson, 2018). In this sense, lagartixas could be excellent models; they are extremely common in tropical cities, have a wide niche breadth, a short generation time (≤3 years: Flower, 1925; Wiederhecker, Pinto, Paiva, & Colli, 2003), and make extreme use of artificial substrates, which are associated with evolutionary change in *Anolis* (e.g., Marnocha et al., 2011; Winchell, Maayan, et al., 2018; Winchell et al., 2016). Further work should explore lagartixas' ecological role in the urban ecosystems and how the selective pressure in cities could affect them.

## CONFLICT OF INTEREST

None declared.

## AUTHORS CONTRIBUTION

I did everything.

## Data Availability

Data already submitted to Dryad: Provisional DOI: https://doi.org/10.5061/dryad.h7t362d
